# Lipomatous Mass Effect on the Brachial Plexus: A Case Report

**DOI:** 10.7759/cureus.81751

**Published:** 2025-04-05

**Authors:** Adrian O Campos, Daniel Blatt, Rehan Zahid

**Affiliations:** 1 Plastic and Reconstructive Surgery, University of Iowa Hospitals and Clinics, Iowa City, USA

**Keywords:** axillary mass, brachial plexopathy, brachial plexus lipoma, brachial plexus neurolysis, peripheral nerve compression, peripheral nerve entrapment

## Abstract

Peripheral nerve compression is a prevalent concern for primary care physicians and hand surgeons, with carpal tunnel syndrome (CTS) and median nerve compression at the wrist being some of the most commonly diagnosed conditions. However, for less common nerve entrapment syndromes, it is crucial for healthcare providers to recognize their symptoms and consider potential underlying issues, particularly those related to the brachial plexus. This case report highlights a 57-year-old male who presented with classic symptoms of left median and ulnar nerve compression in the setting of an enlarging left axillary mass. The patient, a right-hand-dominant male, reported numbness and tingling in the left ulnar-sided digits, as well as weakness in small and ring finger flexion, which began after a fall onto his elbow and outstretched hand a year prior. Initially, the patient experienced significant numbness, tingling, and pain radiating up to the shoulder. Weakness in hand grip, especially affecting the small and ring fingers, and thumb abduction and opposition were also noted. The patient reported transient symptomatic relief with shoulder abduction. Over time, his median nerve compression symptoms improved, with only mild residual tingling noted with shoulder adduction and compression of the axillary mass. However, his ulnar nerve compression symptoms showed minimal improvement despite occupational therapy. The patient had a history of a left axillary lipoma identified five years earlier, which had not been surgically treated, aside from an incisional biopsy that confirmed the pathology. Upon examination, the patient presented with a 7 cm × 10 cm, well-circumscribed, deeply adherent mass in the left axilla. Clinical findings included a positive Wartenberg’s sign of the left small finger, decreased strength in small and ring finger flexion compared to the right, and impaired two-point discrimination of the small and ring fingers. A positive Tinel’s sign was noted at the left cubital tunnel, while the carpal tunnel and Guyon’s canal were negative. Electromyography revealed left-sided ulnar and median nerve compression at the cubital tunnel and carpal tunnel, but could not exclude brachial plexopathy. MRI of the left brachial plexus revealed the lipoma exerting mass effect on the brachial plexus cords and branches, as well as the left axillary vasculature. Surgical intervention involved excision of the left axillary lipoma, brachial plexus exploration and neurolysis, and cubital tunnel release with anterior transposition. Three lipomatous masses were identified, intertwined with the brachial plexus divisions and cords and the axillary vasculature. Meticulous dissection with 3.5× loupe magnification was performed to decompress the brachial plexus. At the six-month follow-up, the patient’s symptoms had completely resolved, and he returned to full activity. This case underscores the importance of evaluating and ruling out brachial plexus pathology in patients presenting with peripheral nerve compression symptoms.

## Introduction

Peripheral nerve compression is a common presentation for primary care physicians and hand surgeons, with carpal tunnel syndrome (CTS) and median nerve compression at the wrist being the most prevalent types [1]. However, for less common nerve entrapment syndromes, it is essential for providers to recognize the presenting symptoms [2]. Regardless of the presentation, it is important to evaluate potential issues related to the brachial plexus. This case report discusses a 57-year-old male who presented with typical symptoms of left median and ulnar nerve compression in the context of an enlarging left axillary mass.

## Case presentation

The patient was a 57-year-old, right-hand-dominant male who presented to the clinic with numbness and tingling in the left ulnar-sided digits, along with weakness in small and ring finger flexion, in the context of an enlarging left axillary mass. The symptoms began after a fall on his left elbow and outstretched hand a year prior to presentation. Immediately following the fall, the patient experienced significant numbness and tingling in his entire left hand, with pain and tingling radiating up to the shoulder. He also experienced weakness in hand grip, particularly in small and ring finger flexion, as well as thumb abduction and opposition. The patient reported transient symptomatic relief with shoulder abduction. Over the course of the year, his median nerve compression symptoms improved, with only mild residual tingling noted with shoulder adduction and compression of the axillary mass. His ulnar nerve compression symptoms showed minimal improvement, with partial improvement in motor strength following occupational therapy.

The patient’s past medical history was notable for a left axillary lipoma identified five years prior, which had not required surgical intervention, aside from an incisional biopsy confirming the pathology.

**Figure 1:**
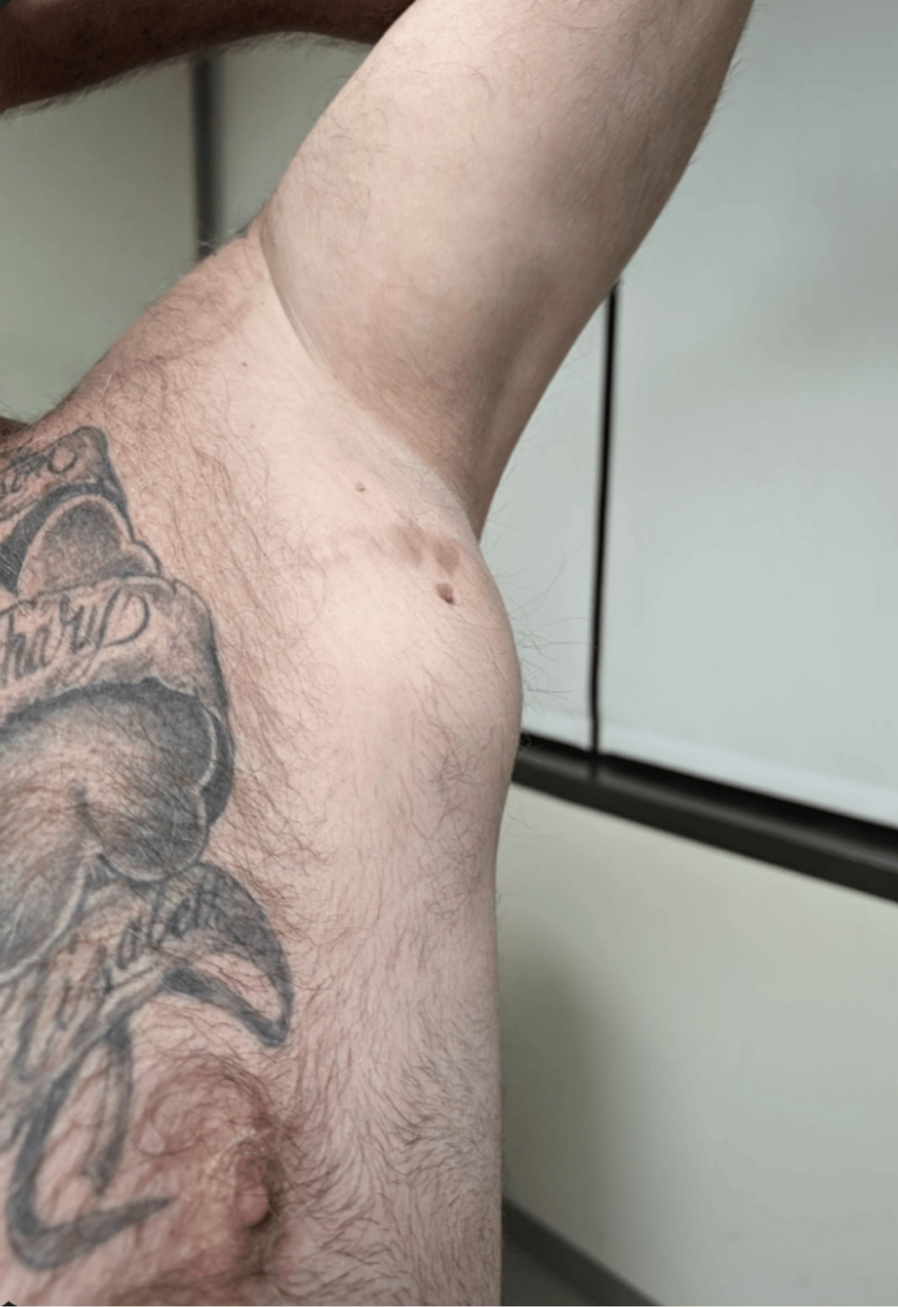
Left axillary mass visualized with the left upper extremity in abduction

On examination, a palpable 7 cm × 10 cm, well-circumscribed, and deeply adherent mass was noted in the left axilla (Figure 1). He had a positive Wartenberg’s sign of the left small finger, decreased strength on flexion of the flexor digitorum profundus in the small and ring fingers compared to the right side, and two-point discrimination of 7 mm in the small and ring fingers. A positive Tinel sign was noted at the left cubital tunnel, but negative at the carpal tunnel and Guyon’s canal.

**Figure 2:**
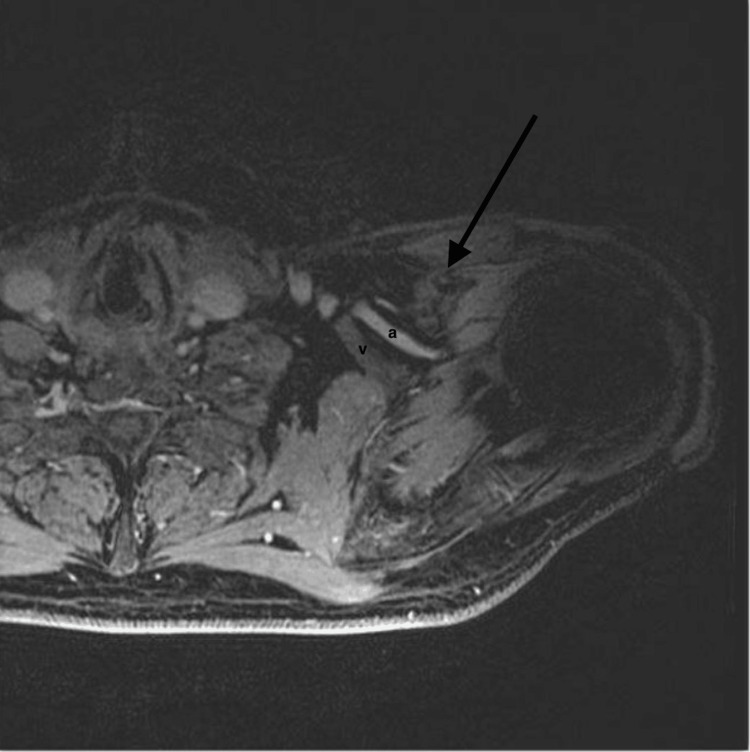
MRI The black arrow points to the left axillary lipomatous mass, showing mass effect on the nearby axillary vasculature (a, v). a, artery; v, vein

An electromyography (EMG) was obtained, which demonstrated left-sided ulnar and median nerve compression at both the cubital tunnel and carpal tunnel. However, the EMG could not exclude a brachial plexopathy. An MRI of the left brachial plexus was also performed, revealing the lipoma with mass effect on the brachial plexus cords and branches, as well as the left axillary vasculature (Figure 2).

Given the patient’s presentation of predominant left-sided ulnar nerve distribution symptoms and the diagnostic evidence indicating that the lipomatous mass in the axilla was contributing with mass effect, a discussion regarding surgical intervention was held. We proceeded with a left axillary lipoma excision, left brachial plexus exploration and neurolysis, and left cubital tunnel release with anterior transposition. The patient did not exhibit clinical evidence of CTS on examination; therefore, a median nerve decompression at the wrist was not performed.

Operative approach

An 8 cm elliptical incision was made surrounding the prior incisional biopsy site within the left axilla. The skin and scar tissue were excised in their entirety. Dissection proceeded through the subcutaneous fat and clavipectoral fascia. Upon reaching the deeper plane, encapsulated tissue suggestive of a lipoma was identified. The lipoma capsule was followed superiorly in deeper, subfascial planes toward the underside of the pectoralis major, tracing the border of the pectoralis minor superiorly. Following this plane, it was found that the lipoma completely engulfed the brachial plexus divisions and cords, as well as the axillary artery and vein.

**Figure 3:**
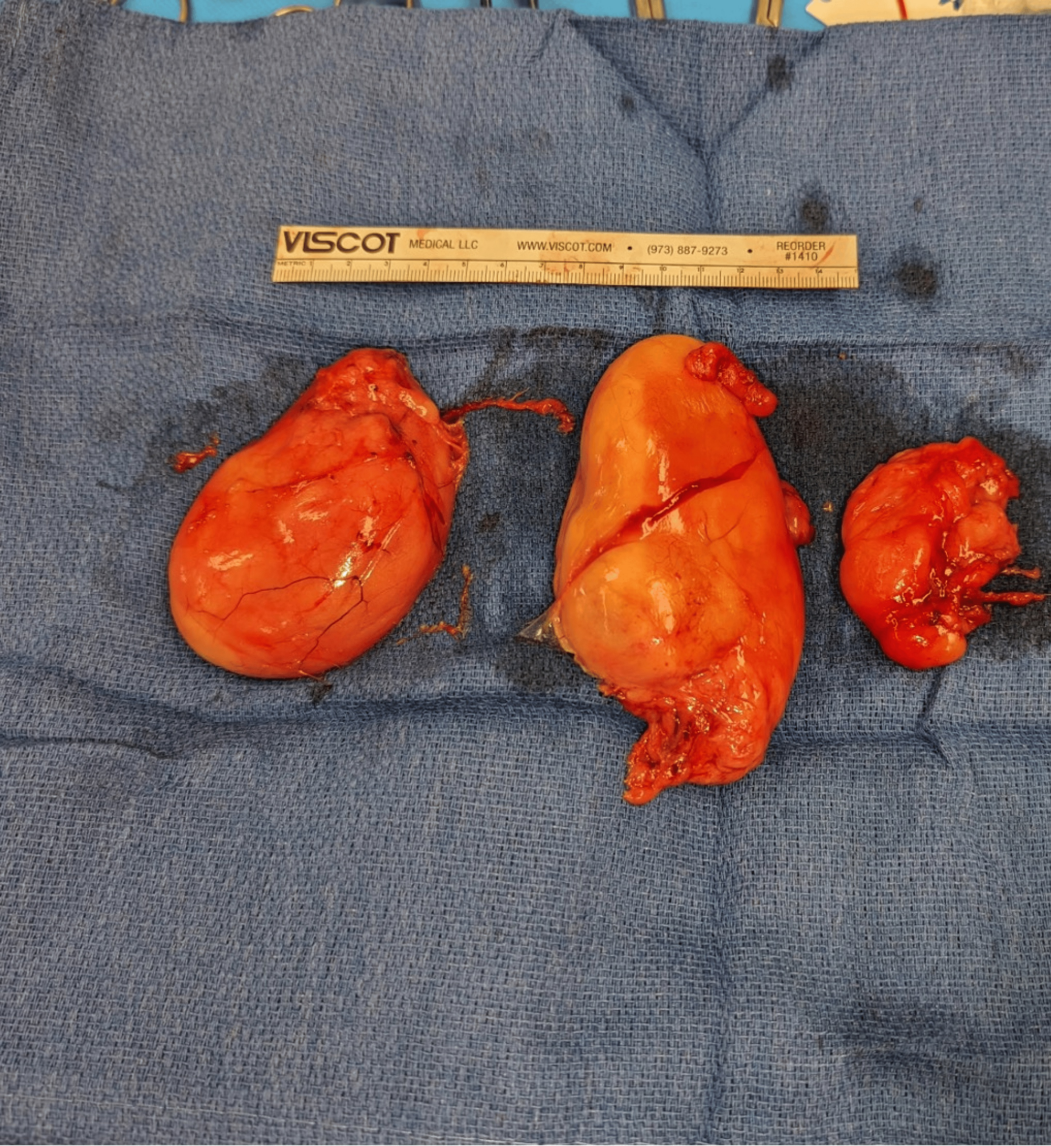
Three lipoma specimens causing mass effect on the brachial plexus

**Figure 4:**
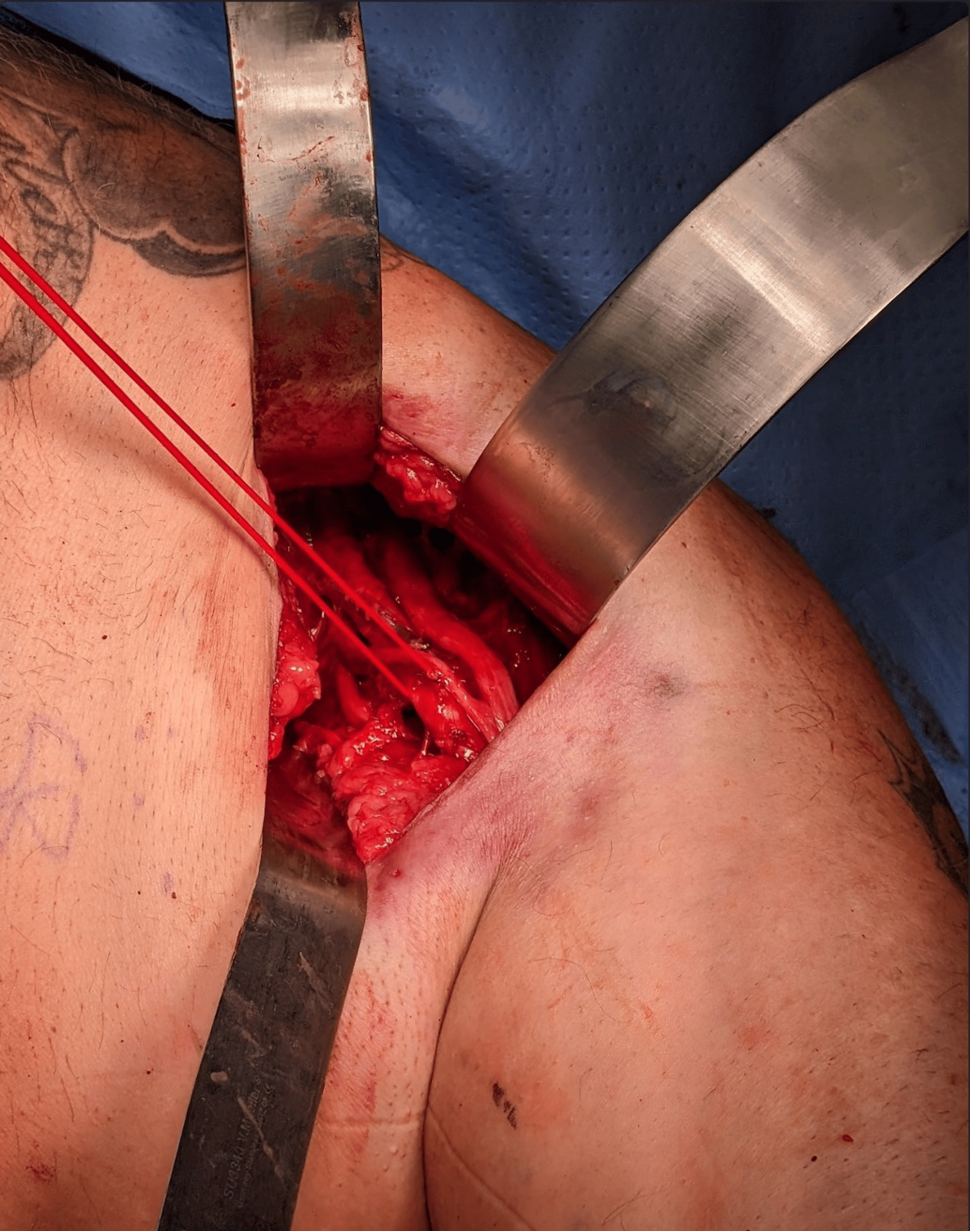
Intraoperative photo of the left axilla A vessel loop encircles the axillary artery, with the more superficial lipoma visualized inferior and posterior to it.

**Figure 5:**
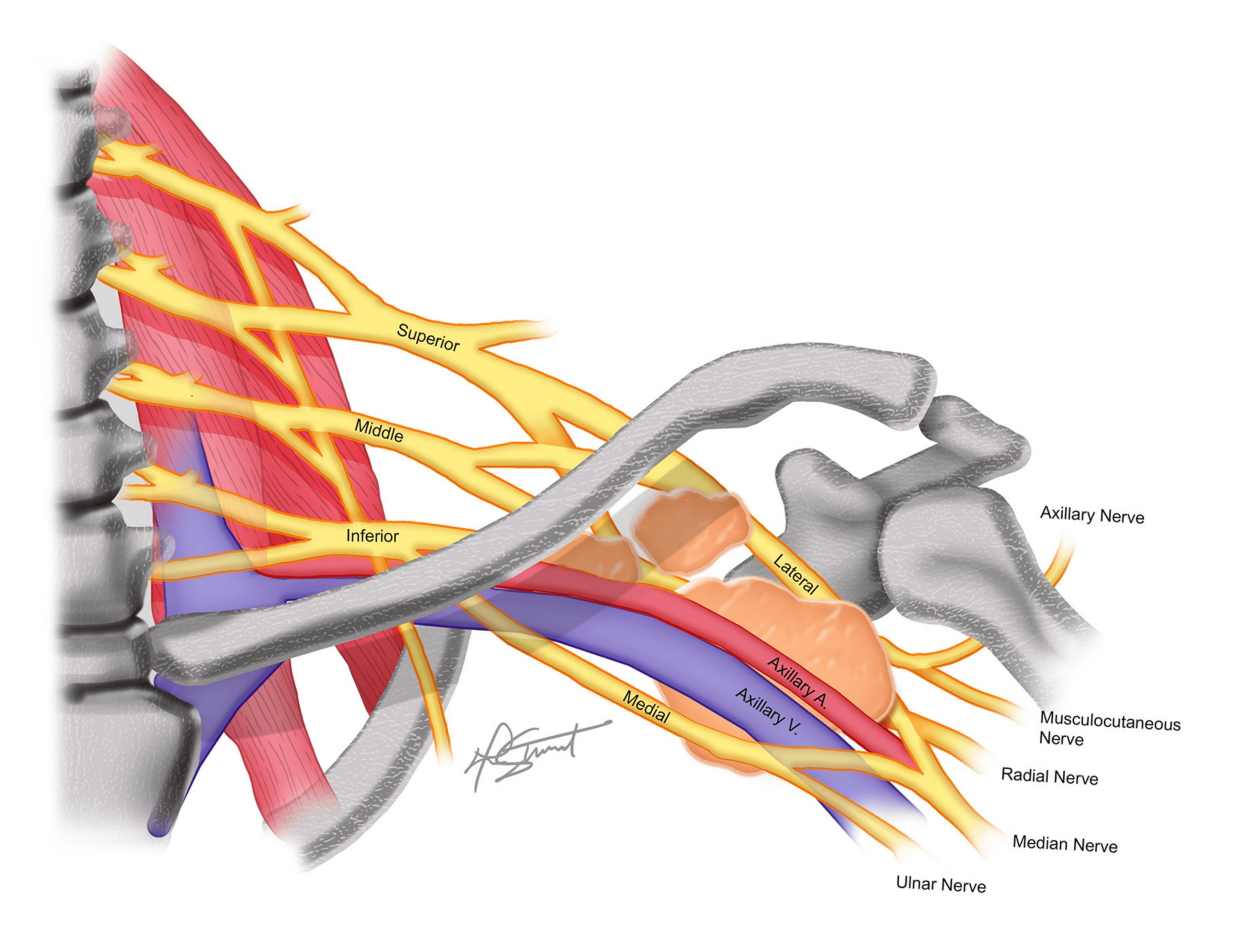
Conceptual illustration of the brachial plexus and axillary vasculature intertwined with the three lipomatous masses Created by Aarin Stewart, Bachelor of Fine Arts, inspired by the work of Hallinan et al. [3].

Meticulous dissection revealed not one, but three lipomatous masses located between the anterior and posterior divisions of the brachial plexus. The more superficial lipoma, measuring 10 cm × 6 cm × 5 cm, displaced the lateral cords superiorly and the axillary artery and vein anteriorly. Just deep to this, two smaller lipomas were identified, measuring approximately 8 cm × 5 cm × 4 cm and 5 cm × 5 cm × 4 cm. Figure 3 shows all three lipoma specimens, while Figure 4 presents an intraoperative view of the left axilla, with a vessel loop encircling the axillary artery and the lipoma visualized inferior and posterior to it. Figure 5 illustrates the complex intertwining of the brachial plexus, axillary vasculature, and lipomas.

Dissection around the axillary artery and vein was performed carefully, always staying along the lipoma capsule to avoid inadvertent injury. The lateral pectoral nerve, medial brachial cutaneous nerve, and antebrachial cutaneous nerve were found to penetrate the larger lipoma and were fully dissected. Once all structures were freed, the lipomas were excised as three separately encapsulated masses, which were sent to pathology. Hemostasis was secured, and a 15-French drain was placed in the axilla. The skin was closed in layers.

Subsequently, cubital tunnel release was performed in the standard fashion. After release, the ulnar nerve was noted to subluxate over the medial epicondyle. A small fascial flap was created to hold the nerve transposed anteriorly. Following hemostasis, the skin was closed in layers, and all incisions were covered with Dermabond. The axilla was then bolstered with gauze to obliterate dead space left by the lipomas. The left arm, shoulder, and chest were wrapped in an ACE bandage to provide compression.

Follow-up

The patient returned to the clinic for a two-week postoperative follow-up. He reported approximately 60% improvement in strength since the surgery and significant relief of the ulnar distribution symptoms in his left upper extremity. Although he still had a positive Tinel sign over the cubital tunnel, this was markedly improved compared to his pre-operative condition. Pathology results indicated lipomatous tissue with benign lymph nodes.

The patient was advised to follow lifting restrictions for five weeks and was scheduled for a three-month follow-up. At the time of his three-month follow-up, the patient canceled his appointment, stating that he had experienced complete resolution of all numbness symptoms and that his strength had returned to normal, now symmetric with the contralateral side. A telephone follow-up was conducted at six months postoperatively. The patient reported having returned to full activity, including heavy weightlifting during exercise, with no reported weakness, numbness, or tingling. Additionally, he noted no recurrence of axillary swelling.

## Discussion

Lipomas are common benign tumors, but lipomas within the brachial plexus are rare. More importantly, brachial plexus lipomas are an uncommon cause of upper extremity nerve compression [4]. In a case series and literature review by Graf et al., only 10 lipomas were resected over a 10-year period (2006-2016) at a single institution [4]. They reported symptom improvement in 80% of patients. Similarly, a systematic review by Gembruch et al. identified 10 articles, comprising a total of 22 brachial plexus lipomas, further demonstrating the rarity of this condition [5].

Preoperatively, based on our clinical examination, we initially expected the lipoma to be positioned anteriorly and superficially to the brachial plexus and axillary vasculature. However, as previously mentioned, the lipomatous masses were intricately situated within the brachial plexus cords and divisions. The axillary vasculature was displaced anteriorly, but careful dissection was still required. Moorefield and Singhal encountered similar cases of multiple-level encasement of the brachial plexus and suggested that microsurgical expertise may be needed for these complex debulking procedures [6]. Our team used 3.5× loupe magnification during the surgery. Given the anatomy of the axilla and the narrow entry point shown in our intraoperative photo (Figure 4), we believe that obtaining clear visualization with a microscope would have been challenging and would have required frequent repositioning.

An interesting aspect of this case is comparing the presenting symptoms with current literature. The predominant presentation of brachial plexus lipomas is thoracic outlet syndrome [7-9]. Our patient, however, presented with distal peripheral nerve compression symptoms consistent with median and ulnar nerve compression. During our clinical exam, we observed a large axillary mass that exacerbated symptoms when the patient adducted his left upper extremity. This, combined with the EMG results showing the inability to rule out brachial plexopathy, led us to diagnose the mass effect of a lipomatous mass on the brachial plexus.

## Conclusions

Overall, brachial plexus tumors are rare, and when encountered, the decision to proceed with surgical intervention can be challenging. As we have experienced, the meticulous dissection required to navigate the complex anatomical structure of the brachial plexus and surrounding vasculature makes the excision of such masses particularly difficult. This case report serves as a reminder to primary care physicians and hand surgeons, who routinely evaluate patients with peripheral nerve entrapment syndromes, to consider and rule out more proximal brachial plexus pathology.

## References

[1] Wright AR, Atkinson RE (2019). Carpal tunnel syndrome: an update for the primary care physician. Hawaii J Health Soc Welf.

[2] Chang J, Neligan P (2023). Plastic Surgery, Volume 6: Hand and Upper Extremity. https://shop.elsevier.com/books/plastic-surgery/chang/978-0-323-81043-2.

[3] Hallinan JT, Pathria MN, Huang BK (2019). Imaging brachial plexus pathology. Appl Radiol.

[4] Graf A, Yang K, King D, Dzwierzynski W, Sanger J, Hettinger P (2019). Lipomas of the brachial plexus: a case series and review of the literature. Hand (N Y).

[5] Gembruch O, Ahmadipour Y, Chihi M (2021). Lipomas as an extremely rare cause for brachial plexus compression: a case series and systematic review. J Brachial Plex Peripher Nerve Inj.

[6] Moorefield AK, Singhal V (2022). Upper extremity mass with lipomatous axillary involvement and multiple level encasement of the brachial plexus. Radiol Case Rep.

[7] Sul J, Lim J, Kang SK (2019). Thoracic outlet syndrome induced by huge lipoma: a case report. Korean J Neurotrauma.

[8] Sergeant G, Gheysens O, Seynaeve P (2003). Neurovascular compression by a subpectoral lipoma: a case report of a rare cause of thoracic outlet syndrome. Acta Chir Belg.

[9] Kuyumdzhiev S, Wall ML, Rogoveanu R (2014). Brachial plexus lipomata presenting with neurogenic and venous thoracic outlet syndrome: case reports and review of the literature. Ann Vasc Surg.

